# Translation activity of chimeric ribosomes composed of *Escherichia coli* and *Bacillus subtilis* or *Geobacillus stearothermophilus* subunits

**DOI:** 10.1016/j.bbrep.2017.05.002

**Published:** 2017-05-12

**Authors:** Sayaka Tsuji, Norikazu Ichihashi

**Affiliations:** a Department of Bioinformatics Engineering, Graduate School of Information Science and Technology, Osaka University, 1-5 Yamadaoka, Suita, Osaka 565-0871, Japan; b Graduate School of Frontier Biosciences, Osaka University, 1-5 Yamadaoka, Suita, Osaka 565-0871, Japan

**Keywords:** Ribosome, *Geobacillus stearothermophilus*, *Bacillus subtilis*, *In vitro* translation, PURE system

## Abstract

Ribosome composition, consisting of rRNA and ribosomal proteins, is highly conserved among a broad range of organisms. However, biochemical studies focusing on ribosomal subunit exchangeability between organisms remain limited. In this study, we show that chimeric ribosomes, composed of *Escherichia coli* and *Bacillus subtilis* or *E. coli* and *Geobacillus stearothermophilus* subunits, are active for β-galactosidase translation in a highly purified *E. coli* translation system. Activities of the chimeric ribosomes showed only a modest decrease when using *E. coli* 30 S subunits, indicating functional conservation of the 50 S subunit between these bacterial species.

## Highlights

• A highly sensitive translation assay was established.
• *B. subtilis* 50S subunit is active for translation in an *E. coli* system.
• *G. stearothermophilus* 50S subunit is active for translation in an *E. coli* system.

## 1 Introduction

Ribosomes play a central role in cellular gene expression. As evidenced by rRNA and ribosomal protein sequence homology, ribosomes are highly conserved among species [1], [2]. The universality and slow substitution rates in rRNA sequences allow for the construction of phylogenic trees of the three kingdoms of life [3], [4]. At a structural level, rRNAs and ribosomal proteins are similar among a broad range of species [5], as shown by the exchangeability of the *E. coli* 16S rRNA gene between distantly related species [6].

In contrast to genetic and structural studies of ribosomes, biochemical studies remain limited. Early studies demonstrated that chimeric ribosomes, composed of *E. coli* and either *B. subtilis* or *G. stearothermophilus* subunits, were active as determined by the poly(U)-dependent poly(Phe) synthesis assay [7], [8], in which the incorporation of phenylalanine in an acid-insoluble fraction is measured using poly-uridine as a template. This assay is, however, not reflective of native protein translation as it does not rely on standard initiation and termination processes. In addition, poly(Phe) synthesis activity is detected even if polymer length is too short to produce a functional protein. Therefore, it remains unclear whether chimeric ribosomes between *E. coli* and other bacterial species are able to produce active proteins [7], [8]. Furthermore, crude *E. coli* extracts were used for the poly-U assay in the previous studies [7], [8]. Therefore, the possible influence of ribosomal proteins and modification enzymes in the extracts cannot be excluded.

In this study, to examine the translation activity of chimeric ribosomes in a controlled environment, we measured the translation of β-galactosidase in a reconstituted *E. coli* translation system. Additionally, the translation activity of the *E. coli* and *B. subtilis* or *E. coli* and *G. stearothermophilus* chimeric ribosomes was determined following further purification.

## 2 Materials and methods

### 2.1 The highly purified translation system

This system consists of all *E. coli* translation proteins except for ribosomes, tRNAs, and low molecular-weight compounds. The system, prepared according to the originally reported method [9], still contained β-galactosidase activity, which was removed through gel-filtration chromatography. The composition was shown in Fig. S1. The preparation methods of the components were reported previously [10]. Each component was purified almost homogeneity as shown in SDS-PAGE data (Fig. S2).

### 2.2 Ribosome purification and subunit preparation

*E. coli* ribosomes were purified as previously described [11]. *B. subtilis* and *G. stearothermophilus* ribosomes were purified following previously reported methods [11], with modifications. Briefly, *B. subtilis* SR22 [12] (kindly provided by Dr. Osamu Makino of Sophia University) was cultured by the same method as *E. coli* [11]. Cells were lysed with a Multi-beads shocker (Yasui kiki, Japan) and ammonium sulfate was added to the supernatant to precipitate protein. The supernatant was applied to a hydrophobic chromatography column. The eluted ribosome fraction was subsequently ultracentrifuged. *G. stearothermophilus* (NBRC 12550 – provided by the National Institute of Technology and Evolution) was cultured by the same method as *E. coli*, except for an increase of incubation temperature to 50 °C. Cells were lysed with a Multi-beads shocker and the lysate was ultragentrifuged. Ribosomal subunits were prepared according to a previous report [13]. Briefly, we performed three rounds of sucrose gradient ultracentrifugation to isolate each subunit for the respective bacterial species.

### 2.3 Translation assay

The reaction solution for the translation assay contains the highly purified translation system, 30–100 nM of each respective ribosome subunit, 10 µM CM-FDG (Life Technologies), 1.75 U/μl T7 RNA polymerase (Takara, Japan), 3.5 nM DNA fragments containing *lacZ*, and 1 U/μl RNase Inhibitor (Promega). The solution was incubated at 37 °C and fluorescence was monitored every 10 min for 15 h with Mx3005P (Agilent Technologies). The maximum rate in fluorescence increase was obtained as the index of translation activity. DNA fragments containing *E. coli* lacZ were prepared by PCR using primers GCGAAATTAATACGACTCACTATAGGG and GGTTATGCTAGTTATTGCTCAGCGG, and pET-lacZ plasmid [14] as template. All experiments were independently carried out three times.

## 3 Results

### 3.1 Highly purified translation assay assessment

**Fig. 1:**
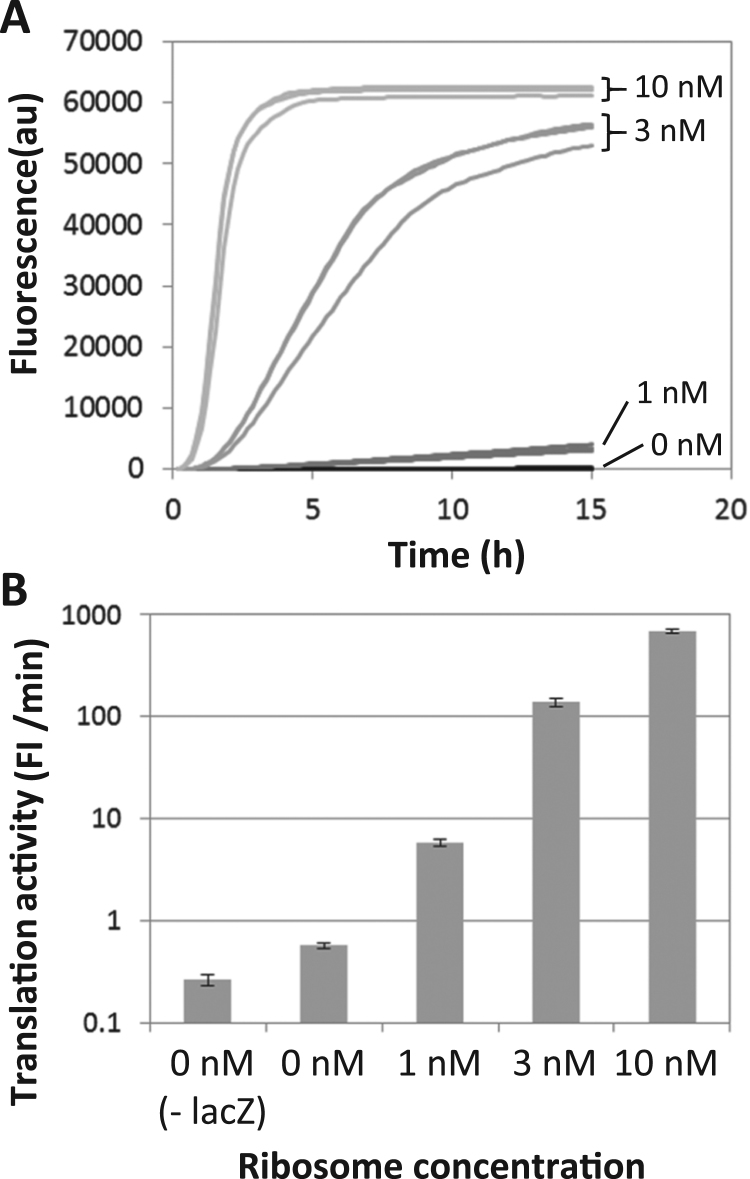
**Ribosome translation activity in a highly purified*****Escherichia coli*****translation system.** A) Time-course data of fluorescence as an indicator of *E. coli* 70S ribosome translation activity. Ribosomes were applied at the indicated concentrations to the β-galactosidase translation assay, as described in the Materials and Methods. Fluorescence produced by the translated β-galactosidase was monitored every 10 min over a total of 15 h. The experiments were performed in triplicate for each ribosome concentration. B) The maximum slope in 1A was plotted as an index of translation activity. The control experiment without lacZ DNA was also performed (- lacZ). The error bars indicate standard deviation (n=3).

A highly sensitive translation assay was first established since the translation activity of a chimeric ribosome under controlled conditions is expected to be too low to detect by standard methods. β-galactosidase was used as the reporter gene as its activity can be measured at single molecule level [15], and the *E.coli* reconstituted translation system [9]. Because the translation system prepared according to the original purification method [9] was contaminated with β-galactosidase activity, we further purified all the components by additional gel-filtration chromatography [10] The sensitivity of this highly purified translation system was tested by adding small amounts of purified 70S *E. coli* ribosome, a DNA fragment containing the β-galactosidase gene, T7 RNA polymerase, and a fluorescent substrate. We measured fluorescence in real-time during incubation at 37 °C for 15 h (Fig. 1A) and obtained the maximum rate in fluorescence increase as an index of translation activity (Fig. 1B), which is reflective of the maximum concentration of β-galactosidase translated. Translation activity in the highly purified system was detected using as little as 1 nM ribosome.

The relationship between ribosome concentration and translation activity is nonlinear at low ribosome concentrations, as shown by replotting the data from Fig. 1B against ribosome concentration (Fig. S2). This is likely to be caused by ribosome adsorption onto tubes or tips during manipulation, which is an issue at very low concentrations of ribosome.

### 3.2 Translation activity of *E. coli* and *B. subtilis* chimeric ribosomes

**Fig. 2:**
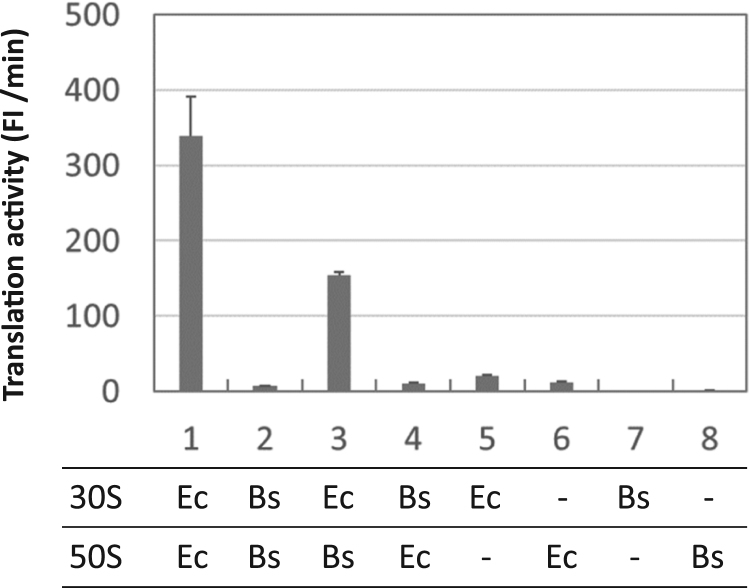
**Translation activity of*****Escherichia coli*****and*****Bacillus subtilis*****chimeric ribosomes.***E. coli* (Ec) and *B. subtilis* (Bs) ribosomal subunits (30 nM) were prepared separately and mixed in the highly purified *E. coli* translation system at the indicated 30S and 50S combinations. The translation activity of each chimeric ribosome was monitored by fluorescence produced by translated β-galactosidase. As an index of translation activity, the maximum rate of fluorescence increase is shown for 10 h reaction time. The error bars indicate standard deviation (n=3). P-values between lanes 1 and 5, 1 and 6, 3 and 4, 3 and 5, and 3 and 8 are <0.03, <0.03, <0.001, <0.001, <0.001, respectively.

To examine the translation activity of *E. coli* and *B. subtilis* chimeric ribosomes, we purified each 70S ribosome and then separated them into their respective 30S and 50S subunits by sucrose gradient ultracentrifugation. The *E. coli* 30S and *B subtilis* 50S subunits were combined and the translation activity of the chimeric protein was measured (Fig. 2). The translation activity of the native *B. subtilis* 70S ribosome in the highly purified *E. coli* translation system (Fig. 2, lane 2) is approximately 1/50th of the native *E. coli* 70S ribosome (Fig. 2, lane 1). This indicates that the *B. subtilis* ribosome has a very minor activity in the *E. coli* translation system, which is consistent with a previous report [16]. Translation activity marginally increased when the *B. subtilis* 50S subunit was substituted by the *E. coli* homolog (Fig. 2, lane 4), while it increased significantly (more than 20-fold) when the 30S subunit was substituted with the *E. coli* homolog (Fig. 2, lane 3, p<0.001). Importantly, the activity levels of the lanes 2 and 4 were higher than the detectable background levels (Fig. 2, lane 5–8, p<0.05). These results demonstrate that chimeric ribosomes, consisting of *E. coli* 30S and *B. subtilis* 50S subunits, are active for β-galactosidase translation in the highly purified *E. coli* translation system.

The translation activity of ribosomes consisting of E. coli 30S and E. coli 50S subunits was significantly lower than that shown in Fig. 1. This is because the translation activity significantly decreases during the dissociation and re-association process of subunits.

### 3.3 Translation activity of *E. coli* and *G. stearothermophilus* chimeric ribosomes

**Fig. 3:**
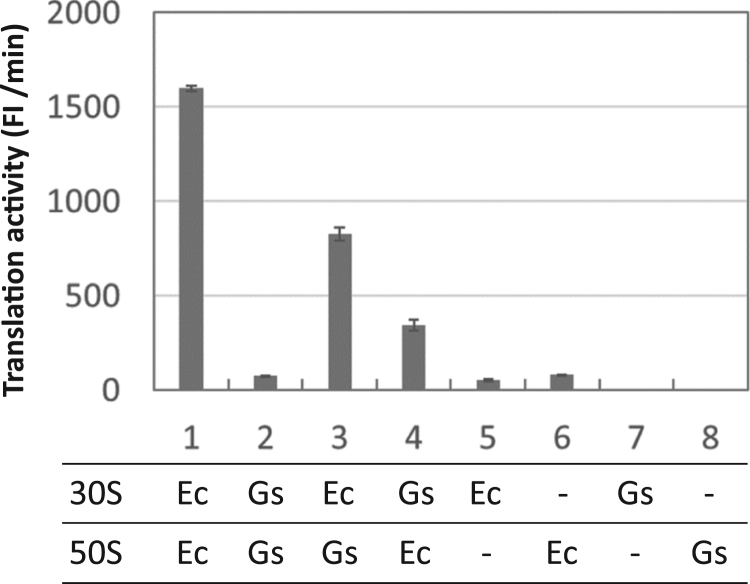
**Translation activity of*****Escherichia coli*****and*****Geobacillus stearothermophilus*****chimeric ribosomes.***E. coli* (Ec) and *G. stearothermophilus* (Gs) ribosomal subunits (100 nM) were prepared separately and mixed in the highly purified *E. coli* translation system at the indicated 30 S and 50 S combinations. The translation activity of each chimeric ribosome was monitored by fluorescence produced by translated β-galactosidase. As an index of translation activity, the maximum rate of fluorescence increase is shown for 10 h reaction time. The error bars indicate standard deviation (n=3). P-values between lanes 1 and 5, 1 and 6, 2 and 7, 2 and 8, 3 and 4, 3 and 5, and 3 and 8 are <0.0001, <0.0001, <0.005, <0.005, <0.001, <0.005, <0.005, <0.01, <0.01, respectively.

The translation activity of *E. coli* and *G. stearothermophilus* chimeric ribosomes was subsequently measured. We purified *G. stearothermophilus* 70S ribosomes and separated them into 30S and 50S subunits by sucrose-gradient ultracentrifugation. The subunits were separately combined with *E. coli* ribosomal subunits and their translation activities were measured (Fig. 3). The translation activity of the native *G. stearothermophilus* ribosome in the highly purified *E. coli* translation system (Fig. 3, lane 2) was approximately 1/20th of the native *E. coli* 70S ribosome (Fig. 3, lane 1). Translation activity increased approximately 10- and 5-fold, respectively, when the *G. stearothermophilus* 30S and 50S subunits were replaced with the *E. coli* homologs (Fig. 3, lane 3 and 4). This result shows that *E. coli* and *G. stearothermophilus* chimeric ribosomes are active for β-galactosidase translation in the highly purified *E. coli* translation system. Similar to the results for *B. subtilis*, the chimeric ribosome had higher activity with the *E. coli* 30S subunit (Fig. 3, lane 3) compared to that with the *E. coli* 50S subunit (Fig. 3, lane 4, p<0.001).

## 4 Discussion

In this study, we demonstrated that chimeric ribosomes between *E. coli* and *B. subtilis* or *E. coli* and *G. stearothermophilus* are active for β-galactosidase translation in a highly purified *E. coli* translation system. For both *B. subtilis* and *G. stearothermophilus*, the chimeric ribosomes incorporating *E. coli* 30S subunits showed only modest decreases in translation activity compared to the native *E. coli* ribosome. This suggests the origin of the 30S subunit is of primary importance to ribosome function in the *E. coli* translation system, while the 50S subunit is exchangeable between bacterial species. This exchangeability of the 50S subunit in the highly purified *E. coli* translation system is consistent with previous reports [7], [8]. However, the previous experiments were performed in crude extracts and therefore the influence of other factors, such as *E. coli* ribosomal proteins or modification enzymes, cannot be excluded. Additionally, poly(U)-dependent poly(Phe) synthesis was the method of measurement in the previous studies, which is not directly comparable to standard protein translation. We therefore performed β-galactosidase translation in a controlled environment, in which all components were purified separately and then combined. Our results verified that the *E. coli* 50S ribosomal subunit could be replaced with either the *B. subtilis* or *G. stearothermophilus* 50S subunit with only a modest reduction in translation activity, suggesting the function of the 50S subunit is conserved among different bacterial species.

We found that the 50S subunit is more exchangeable among species than the 30S subunit. Although we do not know the reason for the difference, it may relate to the role of each subunit. The 30S subunit functions at translation initiation by interacting with initiation factors to recruit the 50S subunit. The recruited 50S subunit provides a platform for translation elongation. The translational elongation mechanisms are highly conserved among species in different domains (e.g., prokarya and eukarya), while initiation mechanisms are less conserved [17], [18]. This might be a reason why the 50S subunit is more exchangeable among species. However, this is a simplified explanation because the role of each subunit cannot be separated clearly. For example, the 50S subunit also has a role in initiation by interacting with IF2 [17]. To understand the difference in exchangeability between the 30S and 50S subunits, further combinatorial experiments using ribosomal subunits and translation factors from different species are needed.

The results of this study can be applied to the complete *in vitro* reconstruction of ribosomes, one of the large challenges in minimal cell synthesis or *in vitro* synthetic biology [19], [20]. The ability of self-reproduction is one of the characteristics of life and has been a target in reconstituting life-like systems [21], [22]. To achieve self-reproduction, all components in the translation system must be reproduced from their corresponding genes. One of the largest challenges is the *in vitro* reconstitution of ribosomes, especially the 50S subunit, which has not been successful in reconstitution from its rRNA gene [13]. The result of the present study indicates that instead of utilizing the *E. coli* 50S subunit, we can use the *G. stearothermophilus* 50S subunit, which has been successfully reconstituted from *in vitro*-transcribed rRNA [23]. This study thus provides another option to achieve the complete *in vitro* reconstitution of ribosomes by utilizing subunits from another bacterial species.
