# Supplementary material for: Translation activity of chimeric ribosomes composed of Escherichia coli and Bacillus subtilis or Geobacillus stearothermophilus subunits
Source: Biochem Biophys Rep. 2017 May 12;10:325–8. doi: 10.1016/j.bbrep.2017.05.002 (PMC5614676; doi:10.1016/j.bbrep.2017.05.002)
Supplement: Supplementary file 2 — Supplementary material [file mmc2.pdf]

Table S1

	Concentration (nM)
IF1	25000
IF2	1000
IF3	4900
EF-G	1100
EF-Tu	80000
EF-Ts	3300
RF1	49
RF2-1	48
RF3	170
RRF	3900
AlaRS	730
ArgRS	31
AsnRS	420
AspRS	120
CysRS	24
GlnRS	60
GluRS	230
GlyRS	86
HisRS	85
IleRS	360
LeuRS	41
LysRS	110
MetRS	110
PheRS	130
ProRS	170
SerRS	78
ThrRS	84
TrpRS	28
TyrRS	150
ValRS	17
MTF	590
MK	1400
CK	250
NDK	16
Ppiase	41
Tig	1000
HrpA	63

Table S1 Composition of the translation system

Figure S1

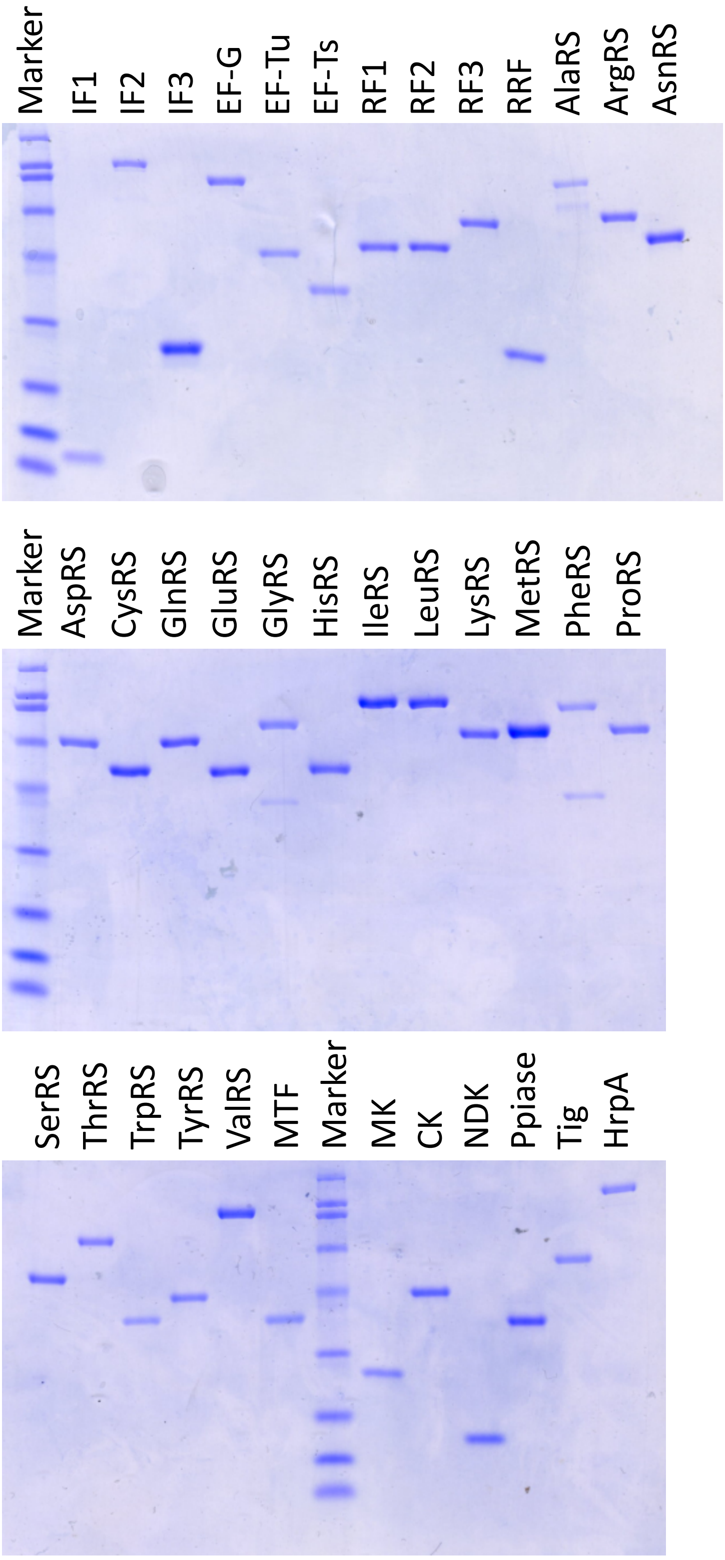

Figure S1 SDS-PAGE of each components of the translation system
Each purified protein (500 ng) was subjected to SDS-PAGE (Any kD Mini-PROTEAN TGX Precast Protein Gels, Bio-Rad Laboratories, Hercules, CA, USA) and stained with Coomassie Brilliant Blue. The size marker contains proteins of 200, 116, 97, 66, 44, 29, 20, 14, and 6.5 kDa.

Figure S2

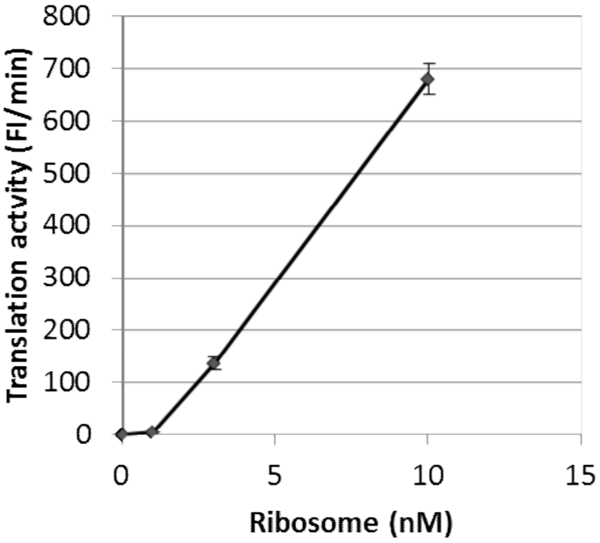

Figure S2 Ribosome translation activity in a highly purified *Escherichia coli* translation system
The data from Figure 1B were replotted against ribosome concentration. The error bars indicate standard deviation (n = 3).
